# Antimicrobial Carboxymethyl Cellulose-Bacterial Cellulose Composites Loaded with Green Synthesized ZnO and Ag Nanoparticles for Food Packaging

**DOI:** 10.3390/ijms252312890

**Published:** 2024-11-30

**Authors:** Iuliana Mihaela Deleanu, Cristina Busuioc, Mariana Deleanu, Anicuţa Stoica-Guzun, Mădălina Rotaru, Vasile Alexandru Ștefan, Gabriela Isopencu

**Affiliations:** 1 Faculty of Chemical Engineering and Biotechnology, National University of Science and Technology “Politehnica” Bucharest, 1-7 Polizu Street, 011061 Bucharest, Romania; iuliana.deleanu@upb.ro (I.M.D.); stefan_alexv@yahoo.com (V.A.Ș.); 2 Institute of Cellular Biology and Pathology “Nicolae Simionescu”, Romanian Academy, 8 Hasdeu Street, 050568 Bucharest, Romania

**Keywords:** bacterial cellulose, carboxymethyl cellulose, turmeric extract, curcumin release, zinc oxide nanoparticles, silver nanoparticles, antimicrobial packaging

## Abstract

Bacterial cellulose (BC) has earned a well-defined place among biopolymers due to its unique physicochemical properties. Unfortunately, native BC lacks antimicrobial and antioxidant properties. To address this limitation, many BC-based nanocomposites with antimicrobial properties have been developed, primarily for applications in the biomedical field, but also for use in food packaging. Many nanoparticles can be incorporated into BC membranes, often in combination with other bioactive molecules. Among the available methods for nanoparticle synthesis, green synthesis has emerged as promising, as it avoids the use of hazardous chemicals. The aim of this paper is to develop and characterize antimicrobial composite materials fabricated using carboxymethyl cellulose (CMC) and bacterial cellulose fibrils loaded with zinc oxide and silver nanoparticles (NPs) obtained using turmeric extract by green synthesis. NP-loaded CMC-BC composites were characterized using scanning electron microscopy (SEM) coupled with energy dispersive X-ray spectroscopy (EDX), attenuated total reflectance-Fourier transform infrared (ATR-FTIR) spectroscopy, Grazing incidence X-ray diffraction (GI-XRD), and thermal analysis (TA). The antibacterial potential of such composites was tested against *Escherichia coli (E. coli)*, *Bacillus subtilis (B. subtilis)*, and *Candida albicans (C. albicans)*.

## 1. Introduction

Bacterial cellulose (BC) is a well-known biopolymer produced through the microbial fermentation of many *Gram-negative* bacterial strains, such as *Komagataeibacter xylinus* (synonyms: *Acetobacter xylinus*, *Gluconacetobacter xylinus*) recognized as one of the most efficient cellulose-producing species. In static culture, BC forms as a gelatinous pellicle at the air-fermentation medium interface and consists of a 3D network structure of nanofibers stabilized by hydrogen bonds [1]. This structure possesses many interesting properties that set it apart from plant cellulose and other biopolymers. These properties, widely reported in the literature, include high crystallinity, a high Young’s modulus, a large specific surface area, and excellent water-holding capacity [2,3]. Other properties that make BC a desirable material for medical, pharmaceutical, cosmetic, food, and food packaging applications are its biocompatibility, non-toxicity, biodegradability, and satisfactory permeability for liquids and gases [3,4,5]. While the intrinsic properties of BC and its composites have tremendous applications, particularly in the biomedical field, interest in their potential as food packaging materials has grown in recent years [6,7]. The development of new food packaging has become an important area of research, as most current products are petroleum-based polymers that are not biodegradable and lead to long-term environmental pollution. The use of biodegradable materials offers a promising solution to this problem. In this regard, cellulose and its derivatives are strong candidates for the food packaging market. The advantage of cellulose is that it can be obtained from many natural sources, including agricultural wastes [8,9]. The cellulose film packaging market is estimated to be valued at US$ 853.1 million in 2024 [10]. In this context, the importance of BC for obtaining food packaging materials has increased, BC being considered one of the finest forms of natural cellulose. It also possesses excellent biodegradability properties and could be transformed into soil fertilizer [11]. However, despite certain limitations, as will be discussed below, native BC shows promise in combination with other materials, including biopolymers and inorganic nanoparticles, to form composites with enhanced properties [7]. For this reason, many scientists are continuing research to develop new composite materials based on BC to be used as food packaging. As for its limitations, it must be emphasized that, in addition to the advantages offered by its properties, BC shows no antimicrobial activity, it lacks biocompatibility for many applications, and after drying BC loses its rehydration capacity. For these reasons, pure BC is less used than BC-biomaterials. In this sense, the great versatility of BC must be recognized. Many techniques were used to obtain BC-composite with nanoparticles (e.g., BC-Ag NP, BC-ZnO, BC-TiO_2_) and BC-biopolymer composites (e.g., dextran, agar, chitosan, starch, collagen, gelatin, sodium alginate) [12,13,14,15]. Another technique to obtain new biomaterials that shows promise is the chemical modification of BC [16]. To transform BC-composites into antimicrobial materials, many techniques have been applied, including using nanoparticles with antimicrobial property deposition, and impregnation of BC-composites with different substances with antimicrobial properties, many of them being plant extracts. Some BC-chitosan composites also show potential antimicrobial properties due to the presence of chitosan [17]. The largest amount of BC used for composite materials is produced in static culture. Static culture offers BC pellicles with high quality but also has many disadvantages, such as low yield, high capital investment, and high operating costs, which can make industrial production of BC economically impractical. The literature is extensive around reducing BC production costs [18,19,20]. Until it is possible to obtain BC inexpensively, a possible solution is to combine BC fibrils with other cheaper biopolymers. From this perspective, carboxymethylcellulose (CMC) is a favorable option. This anionic polymer, which is a derivative of cellulose, is already used in many industrial applications (e.g., cosmetics, food, textiles, detergents, oil drilling, and pharmaceuticals). It has a low cost and high stability. It also shows promise as a biopolymer in the development of new food packaging materials. Some disadvantages that prevent the large-scale use of CMC as food packaging material are its low mechanical strength, brittleness, and hydrophilicity [21]. To overcome these drawbacks, CMC could be blended with other biomaterials such as gelatin, chitosan, collagen, guar gum, polyvinyl alcohol, and sodium alginate, and it could be crosslinked with different substances, most notably citric acid, which is a green alternative to epichlorohydrin and glutaraldehyde [21,22,23,24]. CMC, like BC, lacks the antimicrobial properties essential to an active packaging material. Antimicrobial properties could be conferred upon CMC hydrogels and films by incorporating into their structure antimicrobial agents such as nanoparticles (silver, ZnO, TiO_2_), antimicrobial polymers (e.g., chitosan), or plant extracts with antimicrobial properties [25,26,27,28,29]. Nanoparticles (NPs) are widely used to obtain nanocomposites for active food packaging. From the multitude of metallic nanoparticles, silver and ZnO already enjoy great popularity due to their antimicrobial efficiency against many pathogens. Their incorporation into biopolymers, including BC, can be performed using the different techniques described in the literature [30]. From the multitude of techniques used for nanoparticle synthesis, those of green chemistry enjoy the greatest attention due to the fact that non-toxic chemicals and renewable materials can be used [31,32]. Many plant extracts have been tested in the synthesis of NPs, turmeric extract being only one example [33].

The main component of turmeric extract is curcumin, a natural polyphenolic compound which has proven valuable functional properties. It is widely recognized as a powerful antioxidant, antitumoral, antibacterial, anti-inflammatory, and halochromic agent, with many uses in active and intelligent food packaging technologies [34,35]. Curcumin has been incorporated into various structures, using different techniques, and its release has been investigated in vitro or using real food systems [36]. As sustained release is considered beneficial to extend the shelf life of packaged food, the factors influencing this phenomenon were also investigated. Studies have revealed that polyphenolic compound release could be affected by their concentration in the polymeric matrix, by matrix properties (solubility, erosion, swelling, hydrophobicity), by the diffusion environment (polyphenolics solubility, solvent penetration through polymeric system), and, of course, by the interaction between the compound and the matrix itself [37]. Zinc oxide nanoparticles and curcumin are considered a powerful combination able to improve the physical and functional properties of many composite biomaterials [38].

The aim of this paper is to obtain and characterize antimicrobial composite from CMC and BC fibrils coated with Ag NPs and ZnO NPs using turmeric extract as a reducing agent for NP synthesis. These antimicrobial composites could be applied to the production of food packaging.

## 2. Results and Discussion

### 2.1. Characterization of BC-CMC-NPs Composites

Seven samples were used in this study and their synthesis conditions and symbols are shown in Table 1.

The samples were characterized by scanning electron microscopy (SEM) coupled with energy dispersive X-ray spectroscopy (EDX), attenuated total reflectance-Fourier transform infrared (ATR-FTIR), spectroscopy grazing incidence X-ray diffraction (GI-XRD), and thermal analysis.

Water vapor permeability (WVP) and swelling behavior were also measured.

#### 2.1.1. Scanning Electron Microscopy (SEM) Coupled with EDX

**Figure 1:**
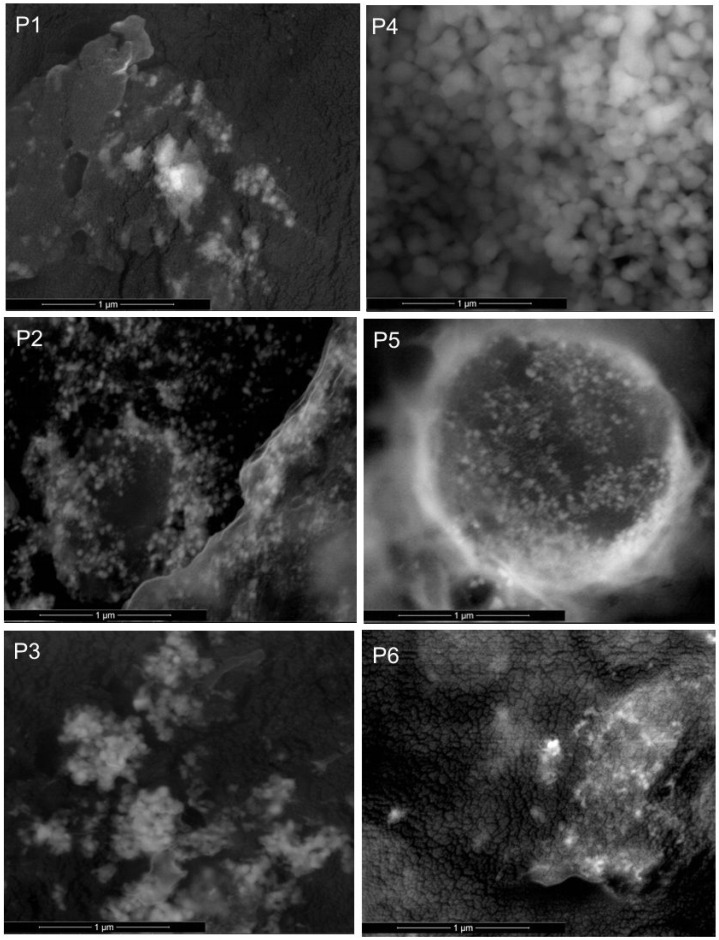
SEM images of P1–P6 samples.

SEM images of the BC-CMC-NP composites obtained and described in Table 1 are presented in Figure 1. In all the images depicted in Figure 1, one can see that NPs are deposited in the CMC-BC biopolymer matrix. If we compare P1 and P4, which contain ZnO NPs deposited under mechanical stirring and under US irradiation, respectively, the ZnO NPs are better distributed in the biopolymer composite under US irradiation (P4 in Figure 1) than those obtained by mechanical stirring (P1 in Figure 1). For P2 and P5 samples, containing Ag NPs, round shaped particles of Ag NPs can be observed. When both Ag and ZnO NPs were deposited on BC fibrils, it became difficult to identify Ag NPs and ZnO NPs, as the particles were joined together on the BC fibrils (P3 and P6 in Figure 1).

The deposition of NPs on BC fibrils was also proved using EDX spectra. The elemental composition of the studied samples is presented in Table 2. The errors are calculated automatically by the software associated with the EDX probe.

If we compare the samples P1 and P4, which contain ZnO NPs, it can be observed that a smaller amount of ZnO was deposited under US irradiation (P4) than under mechanical stirring (P1). In the case of Ag NPs, the situation is different, and under US irradiation the amount of Ag deposited is higher than under mechanical stirring. When both NPS are deposited on BC fibrils it is obvious the Ag NPs are deposited in a larger quantity than ZnO NPs (P3 versus P6).

The observed results can be explained by the effects of US irradiation on particle migration and solubility. Two effects associated with US waves were identified: increased particle instability and the redissolution of smaller particles. These effects arise, on the one hand, from the reduced supersaturation of growth species in the solution and, on the other hand, from intensified high-velocity interparticle collisions. These phenomena are attributed to the acoustic cavitation effects widely described in the literature for obtaining a large number of nanoparticles [39,40], including some deposited on various substrates, such as polyester fiber [41], silk fiber [42], and cotton fabric [43]. In the case of ZnO NPs, the two effects are preventing the formation of larger particles, but in the case of Ag NPs, the opposite is observed, as has previously been reported by others [42,44]. Furthermore, in the co-precipitating systems, nucleation is influenced by the competition between Zn^2+^ and Ag^+^ ions [12].

Analyzing the elemental composition of the samples, as presented in Table 2, it can be seen that the metal concentrations in the final compositions are very low (between 0.97 and 3.4 wt%). In addition, desorption could be influenced by numerous factors, including the characteristics of the food subjected to packaging. In any case, even if the entire amount of metals is desorbed, we can safely assume that it cannot have a negative effect on human cells or the environment, as reported by other studies [45,46].

#### 2.1.2. FTIR Analysis

**Figure 2:**
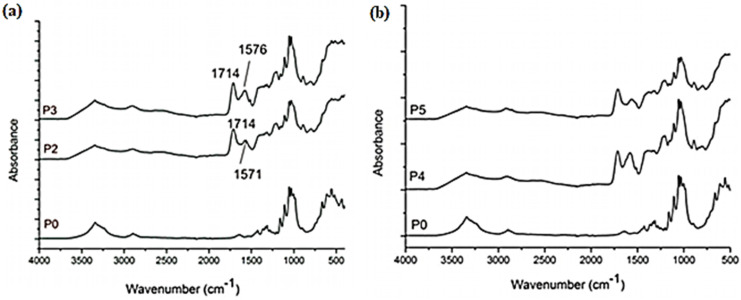
FTIR spectra of samples: (**a**) P0, P2, P3; (**b**) P0, P4, and P5.

The FTIR spectra of studied samples and of the reference sample (P0) are presented in Figure 2. Due to the presence of BC and CMC in the P1–P6 composites, a mixed pattern of bands could be observed. For a better resolution of the spectra, the samples were grouped as follows: P0, P2, and P3 (Figure 2a) and P0, P4, and P5 (Figure 2b). The FTIR spectra of samples P1 and P6 are similar to those of the other samples and, therefore, are not represented in Figure 2.

The broad band at 3340 cm^−1^, corresponding to the stretching frequency of the –OH group, is present in all composites, with slight differences. The main difference between the BC spectrum (P0) and the spectra of P1–P6 samples is the intensity reduction of this band, likely due to interactions between the NPs deposited and –OH groups. The band at 2894 cm^−1^ is attributed to C–H stretching of –CH_2_ and is characteristic of both BC and CMC. This peak can be observed in the spectra of all the studied samples.

Bands at 1032 cm^−1^ and 1159 cm^−1^, attributed to C–O–C stretching vibrations of pyranose ring structure and to C–O–C asymmetric stretching vibration from the glycosidic ring, respectively, could be observed for all the samples. New bands could be observed for all the composites between 1571–1576 cm^−1^ and 1714–1719 cm^−1^. The first band could be assigned to carboxylate asymmetric stretching of Na-CMC, even if there is a displacement in comparison with the value reported in the literature at 1589 cm^−1^. However, considering the composition of the samples P1–P6, there are possible interactions between components which may be responsible for this displacement. The bands between 1714–1719 cm^−1^ can be assigned to the ester bond between anhydride of CA and non-substituted hydroxyl groups of cellulose derivate, which is reported in the range 1715–1735 cm^−1^.

**Figure 3:**
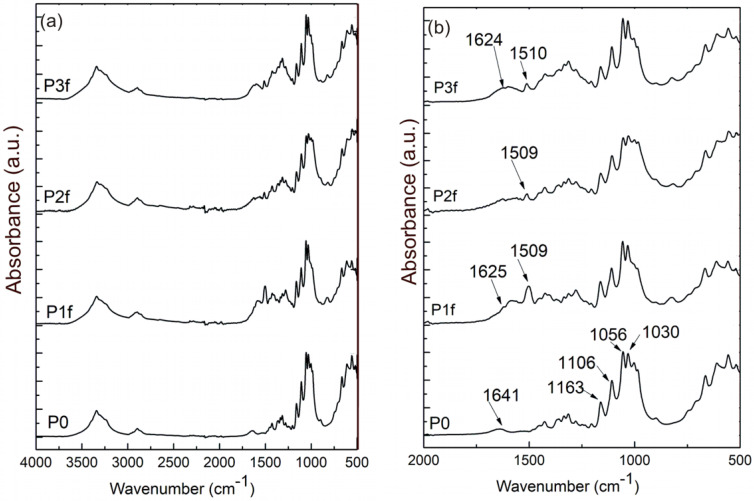
FTIR spectra: (**a**) from 500–4000 cm^−1^ and (**b**) from 500–2000 cm^−1^ for the samples P0 (BC fibrils), P1f (BC fibrils with ZnO NPs), P2f (BC fibrils with Ag NPS), and P3f (BC fibrils with Ag and ZnO NPs).

In order to validate these assumptions, FTIR spectra of BC fibrils with deposited NPs were registered. These spectra are presented in Figure 3. The first observation is that the bands assigned to Na-CMC and to the ester bond between anhydride of CA and non-substituted hydroxyl groups of cellulose derivate are not present in the samples of BC-fibrils with NPs deposited (Figure 3, P1f–Pf3). The band at 1509 cm^−1^ could be assigned to curcumin (C=C stretching vibration) [47,48]. Also, the band at 1641 cm^−1^ which corresponds to water molecules in the amorphous region for BC, is not visible in the studied sample spectra. The bands at 1625 and 1627 cm^−1^ could be assigned to C=O stretching vibration of curcumin molecule. The spectrum of turmeric powder is presented in Supplementary Materials (Figure S1).

#### 2.1.3. XRD

**Figure 4:**
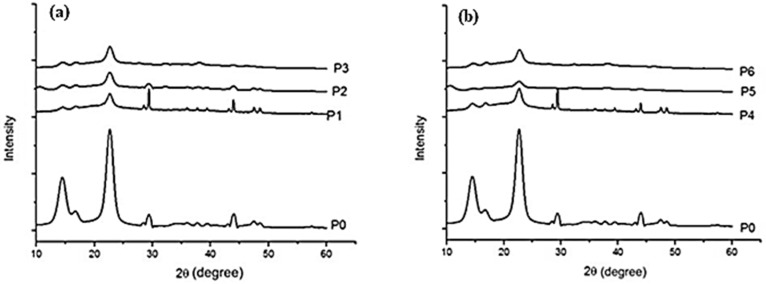
(**a**,**b**) X-ray diffractograms for P0–P6 samples.

Figure 4a,b present the XRD diffraction patterns for the studied samples (P0–P6). All samples exhibit three strong Bragg peaks at 14.4, 16.9, and 22.7 degrees, which correspond to crystalline cellulose I and are characteristic of BC. This observation demonstrates that the crystalline structure of cellulose I was maintained in all the composites. The incorporation of BC fibrils in CMC solution, despite CMC being predominantly an amorphous polymer, does not affect the crystalline structure of BC. The primary differences between the BC spectrum (P0) and the spectra of P1–P6 samples are the reduced intensity of this peak, likely due to interactions between the deposited NPs and the –OH groups [49,50].

#### 2.1.4. Thermogravimetric Analysis

**Figure 5:**
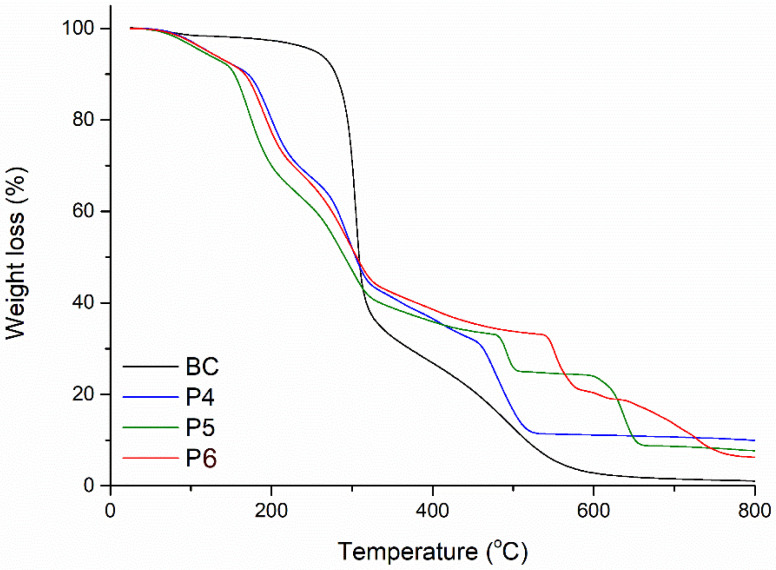
Thermogravimetric analysis (TGA) curves of pure BC and samples P4, P5, and P6.

The thermal behavior of samples P4, P5, and P6 was investigated up to 800 °C in order to assess the loading degree of BC fibrils with inorganic nanoparticles (ZnO and Ag), as well as the possible modifications of BC in terms of thermal stability, triggered by its combination with a metallic or oxide phase, or both simultaneously. For comparison, the thermogravimetric curve of pure BC is included in the graph (Figure 5). The unitary material displays a relatively stable mass up to 200 °C, followed by a pronounced reduction of about 58% in a temperature range of just 120 °C. This continues at a lower rate until a temperature of 600 °C is reached, after which the weight stabilizes reaching the maximum temperature (800 °C), leaving a residual mass below 1%. In contrast, all three composite materials present a similar trend from room temperature to approximately 400 °C, but differ with respect to pure BC, namely, with a continuous decrease with different slopes starting from 80 °C. This can be attributed to the presence of residual water trapped in the material volume, a phenomenon that could be favored by the anchoring of ZnO and Ag NPs at the fibril surface. Moreover, the weight loss occurring above 150 °C could be a result of the dehydration of different hydroxylated species formed during the precipitation process. Beyond 250 °C, the degradation of BC fibrils starts. In a previous study, we considered the influence of turmeric extract by itself, concluding that the extract components shift the main thermal effect to higher temperatures by 10–20 °C, depending on matrix characteristics [51]. In conclusion, the enhancement of thermal stability achieved for the BC composites proposed in this paper represents the complex and synergistic contribution of both precipitated inorganic NPs and impregnated extract compounds. In previous research performed on BC membranes decorated with ZnO NPs by using ammonia as a precipitating agent and ultrasound as a stimulating parameter, the loading degree was estimated at 5 wt.% by thermal analysis means, while the shape of the thermogravimetric curve was quite similar with the one obtained in this study for pure BC [52]. This finding could suggest that the utilization of turmeric extract as a precipitating agent also enhanced the adhesion of ZnO and Ag NPs to the fibrils surface, which subsequently acted as a shield against thermal degradation. Other authors have also reported an increase in the thermal stability of BC when incorporating Ag NPs by a hydrothermal method [53]. In the case of composites, the region of thermal decomposition was shifted right with 60–80 °C relative to pure BC, but still remained below 500 °C; moreover, the process took place in two distinct stages rather than a single one with a wider appearance. Comparing our results, the greater thermostability of the materials obtained in the presence of turmeric extract is obvious, which can be attributed to the improvement of the overall crystallinity of the samples.

### 2.2. Swelling Studies

Swelling ability of a packaging material is of great importance, as it is a clear indicator of water resistance [54]. Also, the quality of the food is strongly affected if water adheres to, or is absorbed by, the packaging film [55].

The results of the swelling study for the samples P1–P6 in distilled water at room temperature are depicted in Table 3. The obtained values are low for all samples, compared to materials based on CMC and/or cellulose, and even compared to those obtained for other polymers with embedded NPs. Existing studies highlight the fact that bacterial cellulose not only has a high tendency to absorb water, but this phenomenon leads to a dramatic deterioration of mechanical properties [56] and it is necessary to reinforce it by adding nanoparticles [57] to the polymer matrix to enhance its malleability after drying [58]. For instance, packaging made of agar-altered foaming BC could exhibit swelling degrees (SD) higher than 95% [59], CMC film alone has a SD of approximately 57% [60], and starch-based films incorporating ZnO could present a swelling index between 27 and 45%, depending on NPs characteristics [61]. In our case, SD values can be correlated with the concentration and type of NPs contained in P1–P6 samples (see Table 2), as NPs are H- bounded to hydroxyl groups of the polymeric matrix, consequently decreasing the free carboxyl groups and water adsorption [54]. It can be concluded that Ag NPs have a stronger influence on water uptake.

### 2.3. Water Vapor Permeability (WVP)

The results of the water vapor transmission rate (WVTR) and water vapor permeability (WVP) tests for the composites P1–P6 are presented in Table 4.

The WVTR values are between 426 and 682 g·m^2^day^−1^, being higher for the samples containing ZnO NPs. As in the case of swelling ability, the presence of ZnO NPs and Ag NPs in the biopolymeric matrix could explain these low values. Previous investigations reported that the addition of NPs strongly affects the moisture barrier properties of films, as the hydrogen bonds that are created increase the adhesion of the matrix and reduce water diffusivity (NPs determines convoluted water trajectory, aggregation induce interstitial gaps, etc.) [61].

For our samples, the NP content correlates strongly with the obtained values for WVTR and WVP. For example, sample P4 has a lower quantity of ZnO NPs in comparison with P1, and has a higher value of WVTR. The same correlation between NP concentration and water resistance was found by Helmiyati et al. (2021) when ZnO NPs were incorporated into CMC-PVA (carboxymethyl cellulose–polyvinyl alcohol) films. The same observation is valid for the samples containing AgNPs. Moreover, the WVTR of films were found between 18–25 g·m^−2^h^−1^, values close to those obtained in this study [54]. In the literature, numerous biodegradable films with BC microfibrils exhibit similar characteristics to those obtained by this study [17].

### 2.4. Curcumin Release Studies

**Figure 6:**
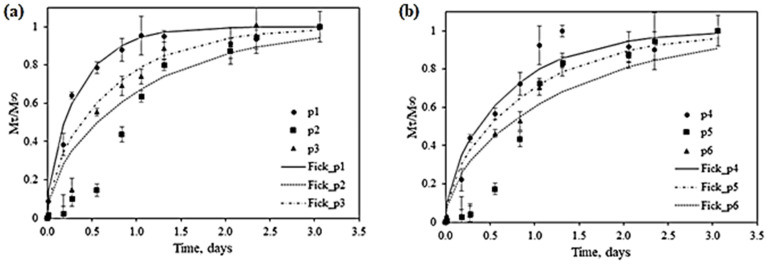
Curcumin release profiles for (**a**) P1–P3 samples and (**b**) P4–P6 samples.

FTIR analyses of the obtained materials showed, the presence of unreacted curcumin. To investigate its release, a preliminary in vitro assessment was conducted, using an ethanolic solution (10% *v*/*v*). The release curves (experimental and theoretical) for all samples are presented in Figure 6a,b.

As can be seen, no significant changes in curcumin release can be attributed to US application, as the time to achieve equilibrium and the concentration dynamics are comparable for similar materials (P1 and P4, P2 and P5, P3 and P6). However, in a basic estimation, considering incipient stages of release investigation, it can be said that curcumin release is mainly influenced by NP type. Thus, P2 and P4, consisting of Ag NPs alone, exhibit the slowest release, particularly in the first 24 h, and the lowest cumulative release overall.

The calculated values of diffusion coefficients, as presented in Table 4, confirm the fastest release for P1 and P4. Furthermore, analyzing the correlation between experimental and theoretical profiles (and the correlation coefficients, presented in Table 4), it can be concluded that when Ag NPs are present, anomalous or non-Fickian transport could be occurring (P2/P3/P5/P6).

There are numerous investigations on curcumin release, and different models (theoretical and empirical) have been employed to describe the behavior and influencing factors, as summarized by Aliabbasi et al., 2021 [34]. As emphasized in many other cases, advanced and detailed investigation on curcumin migration through packaging materials to real food systems are needed. Further investigation is also needed in the present study, to evaluate actual curcumin concentration within each sample, and to correlate it with NPs formation, concentration, and type.

Nevertheless, the preliminary results are encouraging, and prove sustained release of curcumin for three days, as shown by experimental data and by calculated diffusion coefficients, which are similar to data reported by others in similar applications [34].

### 2.5. Antimicrobial Activity

Table 5 presents the inhibition zones for the studied samples.

The obtained results indicate that samples P1–P6 generally show more pronounced antimicrobial activity than those obtained by NP deposition on BC fibrils alone (P1f−P6f). The presence of the cross-linking agent for CMC, citric acid, available in the disc diffusion method, has a significant influence and potentiates the effect of NPs against *E. coli* and slightly against *B. subtilis*. However, the unicellular fungus (*C. albicans*) is not affected by its presence in this form, although it is normally considered a good antibacterial and antifungal agent [62,63]. Samples which contain ZnO NPs (P1 and P4) show significant antimicrobial activity against *E. coli*, followed by *B. subtilis* and moderate activity against *C. albicans*, as is shown in Table 5. It should be noted that the use of US irradiation in the fibril impregnation technology with NPs is a favorable factor for the antimicrobial activity against *E. coli* (20% increase for P4), and against *B. subtilis* (25% increase for P4). If we compare the samples P1 and P4 with those containing only fibrils (P1f and P4f), the antimicrobial activity of the fibrils against *E. coli* is higher. For the samples containing Ag NPs (P2 and P5), the differences between the samples obtained by mechanical stirring in comparison with those obtained by US irradiation are smaller. The antimicrobial activity against *E. coli* and *B. subtilis* is higher for the US-irradiated samples, with increases of 13% and 40.6%, respectively. However, the effect was negative for *C. albicans*, leading to a 57% decrease of the IZ zone. The samples with both NPs deposited on BC (P3 and P6) led to a moderate antimicrobial activity against all tested microorganisms. In this case it seems that the US irradiation had a beneficial influence on antimicrobial activity (P6 versus P3). The samples containing only BC fibrils with both NPs led to a higher activity against *B. subtilis*, the US irradiated sample P6f being superior to P3f. If we compare all the samples with each other, the highest antimicrobial activity against *E. coli* was observed for the sample P6, followed by P4. Against the *B. subtilis* sample, P4 proved the most effective, and against *C. albicans,* the most effective was sample P1. The best antimicrobial activity against all microorganisms studied was obtained for sample P1.

**Figure 7:**
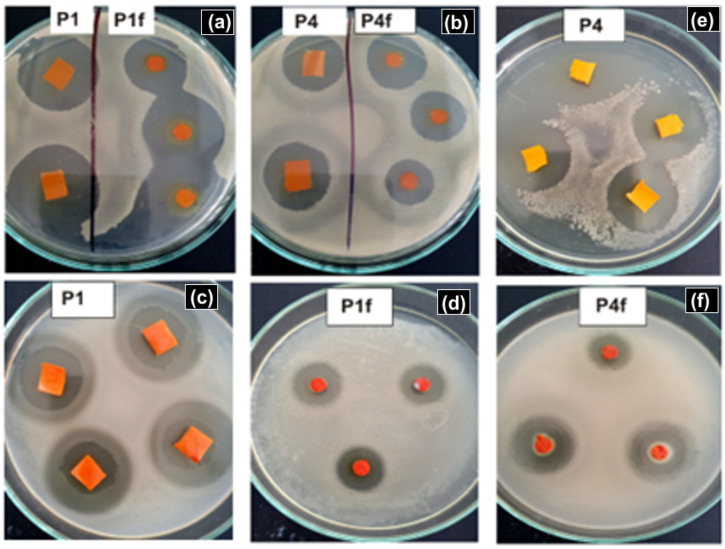
Antimicrobial activity of some representative samples against *E. coli*: (**a**) P1 and P1f, (**b**) P4 and P4f; and against *B. subtilis*: (**c**) P1, (**d**) P1f, (**e**) P4, and (**f**) P4f.

Both NPs deposited on BC fibrils exhibit antimicrobial activity, though their mechanisms of action differ. ForAg NPs, it was demonstrated that the inhibitory potential is due to their physicochemical properties (size and surface) and quantity [64,65]. In this sense, comparing P2 and P5 samples, from EDX analysis samples, P5 contains a higher quantity of Ag NPs (42% increase), and its antimicrobial activity is higher against *E. coli* and *B. subtilis* compared with the P2 sample. The antibacterial action of ZnO is due to the production of reactive oxygen, which results in damage to the cell wall [66,67,68,69]. It is not surprising that the sample with the highest antimicrobial activity (P1) contains the highest quantity of ZnO NPs. A high antimicrobial activity is also demonstrated by sample P4, though it contains fewer ZnO NPs (Figure 7). The lack of activity by the P4 sample against *C. albicans* is difficult to explain, but it could be due to the influence of US irradiation on the crystal synthesis. The cavitation phenomenon creates severe conditions during US irradiation, which influences the nucleation process of ZnO. Therefore, the ZnO crystals could be different from those obtained by mechanical stirring [70,71]. In the case of the samples which contain both NPs (P3 and P6), US irradiation has a positive influence on antimicrobial activity. In this case, P6 has a higher antimicrobial activity, also having a higher quantity of Ag NPs deposited. However, while a synergistic effect between Ag and ZnO NPs was anticipated, it is possible that interactions between Ag and ZnO occur, as Ag NPs have the tendency to bond to the surface of ZnO NPs.

Since turmeric extract was used as reducing agent for both NP syntheses, the influence of curcumin should be also considered. Several studies have demonstrated that curcumin facilitates the diffusion of NPs (Ag and ZnO) into the bacterial cellular membranes, enhancing the antibacterial activity [72,73,74,75].

## 3. Materials and Methods

### 3.1. Materials

All reagents used were of analytical grade (Merck-analytical grade) and all solutions were prepared by using deionized water. Reagents were purchased from Sigma-Aldrich Chemie GmbH (Taufkirchen, Germany): sodium hydroxide (CAS-No-1310-73-2); fructose (Cas-No-57-48-7); sodium carboxymethyl cellulose Mw = 90,000 (CAS-No-9004-32-4); curcumin (CAS-No- 458-37-7); phosphate-buffered saline; citric acid (CAS-No-77-92-9); ethanol (p.a) (CAS-No-64-17-5); YPD Agar; nutrient agar, LB broth, Miller, LB agar, Miller.

Pure organic turmeric powder (*Curcuma longa*) produced in Madagascar was purchased from a local grocery store in Bucharest.

### 3.2. Synthesis of Bacterial Cellulose

The strain employed to produce cellulose was *Gluconacetobacter saccharivorans* isolated from apple cider vinegar. The inoculum was prepared by growing *Gluconacetobacter saccharivorans* at 30 °C using a rotary shaker for 3 days. The inoculum was then transferred to Erlenmeyer flasks of 250 mL in a fermentation medium in a ratio of 1:10. A modified Hestrin-Schramm (MHS) medium containing 2% fructose as a carbon source was used as the fermentation medium. After 7 days at 27 °C in static culture, the pellicles formed at the surface of the fermentation medium were removed and immersed in a 0.5 N NaOH solution at 90 °C to remove bacterial cells from the BC membrane. The pellicles were then rinsed several times with distilled water, until the water pH became neutral and the impurities were fully removed. The BC pellicles were then stored in distilled water at 4 °C.

### 3.3. Synthesis of Ag NPs and ZnO NPs

The turmeric extract was prepared using food-grade ethanol in a Soxhlet apparatus over a period of 6 h. After extraction, the solution was filtered and analyzed to determine its curcumin content using an UV-VIS spectrometer (UniSpec2 Spectrophotometer, LLG-Labware, Meckenheim, Germany) at a fixed wavelength of 429 nm, following a standard curcumin calibration curve. Turmeric extract with a curcumin content of 4.5 M was used as a reduced agent for NP synthesis. The NPs were synthesized on BC fibrils, obtained after grinding BC pellicles with a high-speed grinder. The fibrils were partially squeezed, with a final moisture content of 96.1%. For ZnO NP synthesis, a co-precipitation method was used, with 100 mL zinc acetate solution (0.1 M) as the precursor. Wet BC fibrils (10 g) were dispersed under mechanical stirring for five minutes. Then, 50 mL turmeric extract was added, and the mixing was continued for another 5 min. The BC fibrils with deposited ZnO NPs were separated by filtration from the suspension, partially squeezed, and dispersed in a CMC aqueous solution (0.8% *w*/*w*) containing citric acid (1:1 mass ratio CMC:CA). After vigorous stirring for 1 h, the dispersion was poured into Petri dishes and dried for 6 h at 45 °C in a food dryer, before being subjected to 80 °C for 10 min for curing.

For Ag NPs deposition on BC fibrils, the same method was used as for ZnO NPs, with the difference that 100 mL AgNO_3_ (0.1 M) was used as precursor substance. Turmeric extract once again served as the reducing agent. When both NPs (Ag and ZnO) were deposited on BC fibrils, these were dispersed in 50 mL zinc acetate solution (0.1 M) and 50 mL AgNO_3_ (0.1 M) under vigorous stirring for 5 min, the mass of BC wet fibrils being 10 g. After the dispersion of BC fibrils, 50 mL of turmeric solution was added and stirring was continued for 5 min. The final suspension was filtered and the BC fibrils with Ag NPs and ZnO NPs were dispersed under mechanical stirring in a CMC solution (0.8% *w*/*w*) containing citric acid (mass ratio between CMC:CA being 1:1). After vigorous stirring for 1 h, the dispersion was poured into Petri dishes and dried for 6 h at 45 °C in a food dryer and then subjected to 80 °C for 10 min for curing.

To investigate the influence of US irradiation on NP synthesis, the second part of the experiment involved dispersing 10 g wet BC fibrils in precursor solutions under US irradiation at 40% probe power using an Ultrasonic Processor VCX 500 (Sonics & Materials, Inc., Newtown, CT, USA), working at 500 W and 20 kHz. First the BC fibrils were dispersed for 5 min in 100 mL zinc acetate solution (0.1 M) or in 100 mL AgNO_3_ (0.1 M), and then the turmeric extract (50 mL) was gently poured into the reaction vessel, and the contact time was maintained (also 5 min). For simultaneous Ag and ZnO NPs synthesis, the BC fibrils were dispersed in 50 mL zinc acetate solution (0.1 M) and 50 mL AgNO_3_ (0.1 M) under US irradiation for 5 min, followed by turmeric extract addition and US irradiation for another 5 min. The final suspensions were filtered and the BC fibrils with NPs deposited were dispersed under mechanical stirring in a CMC solution (0.8% *w*/*w*) containing citric acid (1:1 mass ratio CMC:CA). After vigorous mechanical stirring for 1 h, each dispersion was poured into Petri dishes and dried for 6 h at 45 °C in a food dryer before being subjected to 80 °C for 10 min for curing.

**Figure 8:**
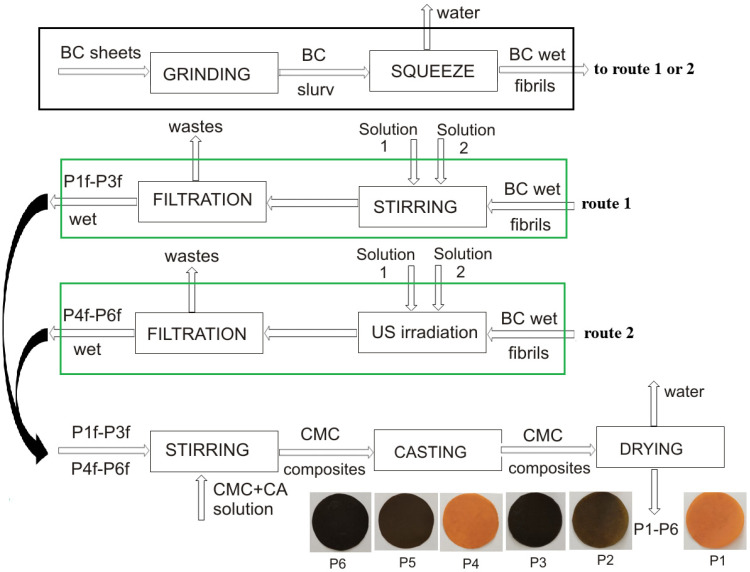
Schematic representation of the sample preparation.

Figure 8 shows a schematic representation of the BC processing method before its mixture with Solution 1 (0.1 M zinc acetate solution) and Solution 2 (turmeric ethanolic extract), respectively, processed in two routes (route 1—with mechanical mixing and route 2 with US) and then embedded in the CMC polymer matrix.

### 3.4. Characterization of Composites P0–P6

The obtained samples were characterized from morphological, structural, compositional, and thermal points of view. The morphology was investigated by scanning electron microscopy (SEM) with an FEI Quanta Inspect F50 microscope (Thermo Fisher Scientific, Waltham, MA, USA) equipped with an energy-dispersive X-ray spectroscopy (EDX) probe; the working distance was set at 10 mm and an accelerating voltage of 20 kV was used, the sample surfaces being coated with a thin layer of gold by DC magnetron sputtering for 40 s. The chemical bonds and groups were studied through attenuated total reflection-Fourier transform infrared (ATR-FTIR) spectroscopy with a Nicolet iS50 spectrophotometer (Thermo Fisher Scientific, Waltham, MA, USA), in the wavenumber range 400–4000 cm^−1^, 4 cm^−1^ resolution, and 64 scans/sample. The crystal structure and phase composition were studied by X-ray diffraction (XRD) with a PANalytical Empyrean diffractometer (Malvern Panalytical, Almelo, The Netherlands), using Ni-filtered Cu Kα radiation (λ = 1.54 Å), in the 2θ range 10–60°, 2 °/min scan speed, 0.02° step size and 0.6 s preset time.

### 3.5. Swelling Studies

To determine the SD, 2 cm × 2 cm square film pieces were dried to constant weight and immersed in distilled water at ambient temperature (20 °C). SD was calculated by measuring the initial weight, m_i_ (g), and the weight of the swollen samples, m_s_ (g), using the Equation (1):

(1) $$SD\;(\%)=\left(m_{s}-m_{i}\right)\cdot 100/m_{i}$$

Since the observed time necessary to achieve equilibrium was very brief (approx. 30 min), any material loss due to dissolution or diffusion was neglected.

### 3.6. Water Vapor Permeability

Water vapor permeability was determined for all composite membranes. The procedure followed the method earlier described by [76]. Three samples for each material were cut to a diameter of 3 cm and individually sealed in small cups containing the same amounts of dried silica gel (0% RH). The sealed cups were then placed in a desiccator containing distilled water. The absorbed moisture was determined by periodic gravimetric measurements of the cups, at room temperature (25 °C) and 98% relative humidity (measured with a humidity sensor). The final measurements were completed after 5 days (120 h) of incubation. WVP was determined according to the Equation (2) [77]:

(2) WVP=WVTR·δ/Δp

where: WVP represents water vapor permeability (g m^−1^ s^−1^ Pa^−1^), *WVTR* is the water vapor transmission rate through the membrane (g m^−2^ h^−1^), δ is the membrane thickness (m), and Δp represents the water vapor partial pressure difference across the two sides of the membrane (Pa).

Composite thickness was measured to the nearest 1 μm with a laboratory micro-meter (Mitutoyo Mfg Co. Ltd., Kawasaki, Japan). The average value of three measurements was used for further calculations.

### 3.7. Kinetics of Curcumin Release and Theoretical Evaluation

The total immersion assay technique was used to study the unreacted curcumin release from the composites P1–P6. Square pieces of 1 cm^2^ from each film were cut and immersed in 50 mL of an ethanol-distilled water (10:90, *v*/*v*) solution. The samples were shaken at room temperature at 100 rpm on an orbital shaker (LBX Orb-Pro, Labbox Labware, S.L., Barcelona, Spain). Periodically, 1 mL of the solution was removed to determine curcumin content. To maintain the sink condition, fresh ethanol solution was added after each determination. A UV-VIS spectrometer (UniSpec2 Spectrophotometer) was used to measure curcumin presence at a fixed wavelength of 429 nm, according to the standard curcumin calibration curve.

The experiments were performed in triplicate and the average values were reported.

To examine the curcumin release, Fick’s diffusion model, based on the second law of diffusion, was applied. Hypothesizing that films are thin, homogenous plane sheets, and that curcumin is uniformly distributed within it, the amount of curcumin released at time τ is given by Equation (3):

(3) $$M_{Fickian}\left(\tau \right)=M_{\infty }\cdot \left(1-\frac{8}{\pi^{2}}\sum_{n=0}^{\infty }\frac{1}{(2n+1{)}^{2}}\cdot \exp\left(-\frac{(2n+1{)}^{2}\pi^{2}}{4\delta^{2}}\cdot D\cdot \tau \right)\right)$$

where: *D* represents the diffusion coefficient (m^2^ s^−1^), $M_{Fickian}$ represents the absolute cumulative amount of curcumin released from film at time τ by Fickian diffusion, $M_{\infty }$ is the absolute cumulative amount of curcumin released at infinite time (equilibrium), *n* is the summation index, δ is the film thickness (m), and τ is the release time (s).

The diffusion coefficients were calculated using Equation (4), valid for early stages of diffusion $\left(M_{\tau }/M_{\infty }<2/3\right)$ [78].

(4) $$D={\left(\frac{k^{\prime }\cdot \delta }{4}\right)}^{2}\pi$$

where: $k^{\prime }$ is the slope of the linear regression of $M_{\tau }/M_{\infty }$ versus $\tau^{1/2}$.

### 3.8. Antimicrobial Activity

The antimicrobial activity of samples P1–P6 and of control samples were investigated using the adapted disc diffusion assay. Measurements were made both for BC fibrils on which NPs were deposited, before being embedded in CMC solution and cross-linked with CA, as well as for the final samples with CMC-BC loaded with NPs. BC-NPs samples prior to incorporation into CMC were named P1f (BC-ZnO NPs), P2f (BC-Ag NPs), P3f (BC-Ag-ZnO NPs), P4f (BC-ZnO NPs/US), P5f (BC -Ag NPs/US), and P6f (BC-Ag-ZnO NPs/US). The microorganisms used for the study were: *Escherichia coli* (as Gram-negative bacteria), *Bacillus subtilis* (as Gram-positive bacteria), and *Candida albicans* (as pathogenic fungus). Every microorganism was cultivated on specific media, purchased from Sigma-Aldrich, such as: LBA (Luria Bertani agar) for *E. coli*, NA (Nutrient Agar) for *B. subtilis*, and YEPD (Yeast Extract Peptone Dextrose) for *C. albicans*. The samples were cut into disc shapes with a diameter of 6 mm or square shapes with a side of 6 mm. The samples were sterilized by UV (254 nm) for 30 min for each side. The inoculum for each strain was adjusted to correspond to 0.5 McFarland Standard concentration measured at 600 nm. The 100 μL of inoculum for each strain was spread in Petri plates and was pipetted on the surface of the solidified culture medium. The sample disks were placed aseptically in the plates and were incubated at 37 °C for 24 h, after which the inhibition zone was measured (IZ, mm).

The statistical analysis was performed using one-way analysis of variance (ANOVA) (*p* < 0.05) and post-hoc testing based on the studentized range distribution, Tukey’s HSD to identify significant differences among the samples. The sample size was three (experiments were performed in triplicates).

## 4. Conclusions

The aim of this study was to propose new composite materials containing bacterial cellulose fibrils, CMC, zinc oxide, and silver nanoparticles (NPs) obtained using turmeric extract by green synthesis, with potential applications in food packaging. SEM images showed that NPs were deposited within the CMC-BC biopolymer matrix, and ultrasound irradiation influenced NP distribution in the polymer matrix. Elemental composition analysis of the studied samples using EDX showed that US irradiation also has an influence on the amount of NPs deposited in the composites, the quantity of ZnO NPs deposited being smaller under US irradiation, and that of Ag NPs being larger under US irradiation. FTIR spectra confirmed strong compatibility between CMC and BC, and the presence of unreacted curcumin from the turmeric extract used for NP synthesis. For this reason, a study of the curcumin released was also conducted, concluding that curcumin release is mainly influenced by NP type. More in-depth studies should be conducted to explain this phenomenon. Water vapor permeability, which is an important characteristic for a food packaging material, decreased for the composites containing Ag NPs and Ag/ZnO/Nps, regardless of the obtainment method. The highest antimicrobial activity was observed for the samples containing ZnO NPs.

These results support the potential of the studied composites as strong candidates for antimicrobial food packaging applications. This study is the first step in an investigation that could proceed in at least two directions: first, to test the new composites for their antimicrobial effects using a model food, and second, to test the biodegradability of these composites in soil.

## Figures and Tables

**Table 1:** Sample composition matrix and sample symbol.

Sample Abbreviation	CMC	BC Fibril	ZnO NPs	Ag NPs	US Use
P1	+	+	+	−	−
P2	+	+	−	+	−
P3	+	+	+	+	−
P4	+	+	+	−	+
P5	+	+	−	+	+
P6	+	+	+	+	+
P0	−	+	−	−	−

BC—bacterial cellulose; CMC—carboxymethyl cellulose; US—ultrasound.

**Table 2:** Elemental composition of the P0–P6 samples using EDX analysis.

Sample	Element	wt%	at%	Error, %	Sample	Element	wt%	at%	Error, %
P0	C K	48.69	55.83	7.03					
	O K	51.31	44.17	11.53					
P1	C K	41.70	49.19	7.66	P4	C K	38.54	45.81	7.93
	O K	57.07	50.54	10.88		O K	60.49	53.98	10.69
	Zn K	1.23	0.27	20.45		Zn K	0.97	0.21	22.11
P2	C K	37.29	45.01	8.09	P5	C K	38.04	46.18	7.93
	O K	60.33	54.67	10.82		O K	58.55	53.36	10.92
	Ag L	2.38	0.32	20.67		Ag L	3.40	0.46	16.3
P3	C K	38.07	45.84	8.06	P6	C K	38.52	46.41	8.06
	O K	59.48	53.76	10.61		O K	58.77	53.16	10.78
	Ag L	1.68	0.22	27.56		Ag L	1.99	0.27	23.33
	Zn K	0.77	0.17	23.73		Zn K	0.71	0.16	29.74

**Table 3:** Swelling ratio of the studied sample: P1–P6.

Sample	SD, %
P1	30.37 ± 1.82
P2	6.56 ± 1.15
P3	6.74 ± 0.52
P4	19.70 ± 1.74
P5	9.33 ± 1.38
P6	6.99 ± 1.22

**Table 4:** Measured and calculated parameters for P1–P6 samples: water vapor transfer rate, water vapor permeability, film thickness, and the diffusion coefficients together with correlation coefficients.

Sample	WVTR (g·m^−2^day^−1^)	WVP (g·m^−1^s^−1^Pa^−1^) × 10^10^	Thickness (mm)	D (m^2^s^−1^) × 10^14^	R^2^
P1	676.4 ± 8.750	3.362 ± 0.100	0.144 ± 0.140	5.620 ± 0.360	0.987
P2	455.4 ± 6.380	2.959 ± 0.078	0.183 ± 0.007	2.670 ± 0.780	0.910
P3	600.0 ± 12.100	2.987 ± 0.045	0.140 ± 0.005	2.830 ± 0.710	0.940
P4	682.3 ± 12.280	3.827 ± 0.040	0.158 ± 0.019	3.130 ± 0.280	0.964
P5	426.0 ± 6.816	2.012 ± 0.032	0.133 ± 0.003	2.050 ± 0.520	0.903
P6	559.5 ± 6.714	2.920 ± 0.075	0.147 ± 0.038	2.880 ± 0.650	0.928

The parameters are presented as mean values ± standard deviation (n = 3).

**Table 5:** Inhibition zones for analyzed samples.

Sample Code	IZ, mm ± Standard Deviation Mean		
	*E. coli*	*B. subtilis*	*C. albicans*
P1	5.00 ± 0.67	12.00 ± 1.70	5.00 ± 0.42
P1f	9.33 ± 0.86	6.44 ± 0.54	4.25 ± 0.33
P2	5.00 ± 0.47	3.11 ± 0.21	2.50 ± 0.22
P2f	2.56 ± 0.24	1.11 ± 0.67	3.50 ± 0.22
P3	5.56 ± 0.48	4.33 ± 0.67	1.00 ± 0.24
P3f	2.67 ± 0.38	5.22 ± 0.78	2.50 ± 0.22
P4	6.00 ± 0.33	15.00 ± 1.50	nd
P4f	6.50 ± 0.22	7.00 ± 0.56	0.67 ± 0.03
P5	5.67 ± 0.24	4.22 ± 0.53	1.44 ± 0.37
P5f	2.83 ± 0.07	1.67 ± 0.15	3.00
P6	7.00 ± 0.87	4.78 ± 0.56	2.33 ± 0.33
P6f	4.83 ± 0.27	6.11 ± 0.98	1.33 ± 0.06
P0	nd	nd	nd
CMC/CA	3.50 ± 0.58	1.83 ± 0.24	nd

nd—non detected.

## Data Availability

Data is contained within the article or Supplementary Materials.

## Supplementary Materials

The following supporting information can be downloaded at: https://www.mdpi.com/article/10.3390/ijms252312890/s1.
