# Supplementary material for: Antimicrobial Carboxymethyl Cellulose-Bacterial Cellulose Composites Loaded with Green Synthesized ZnO and Ag Nanoparticles for Food Packaging
Source: Int J Mol Sci. 2024 Nov 30;25(23):12890. doi: 10.3390/ijms252312890 (PMC11641031; doi:10.3390/ijms252312890)
Supplement: Supplementary file 1 [file ijms-25-12890-s001.zip › ijms-3315796-supplementary.pdf]

Antimicrobial Carboxymethyl Cellulose-Bacterial Cellulose Composites Loaded with Green Synthesized ZnO and Ag Nanoparticles for Food Packaging

Iuliana Mihaela Deleanu[1], Cristina Busuioc[1], Mariana Deleanu[2], Anicuţa Stoica-Guzun[1], Mădălina Rotaru[1], Vasile Alexandru Ștefan[1], Gabriela Isopencu[1,*]

[1] National University of Science and Technology "Politehnica" Bucharest, Faculty of Chemical Engineering and Biotechnology, 1-7 Polizu Street, 011061 Bucharest, Romania

[2] Institute of Cellular Biology and Pathology "Nicolae Simionescu" of the Romanian Academy, 8 Hasdeu Street, 050568 Bucharest, Romania

* Correspondence: gabriela.isopencu@upb.ro

Supplementary Material

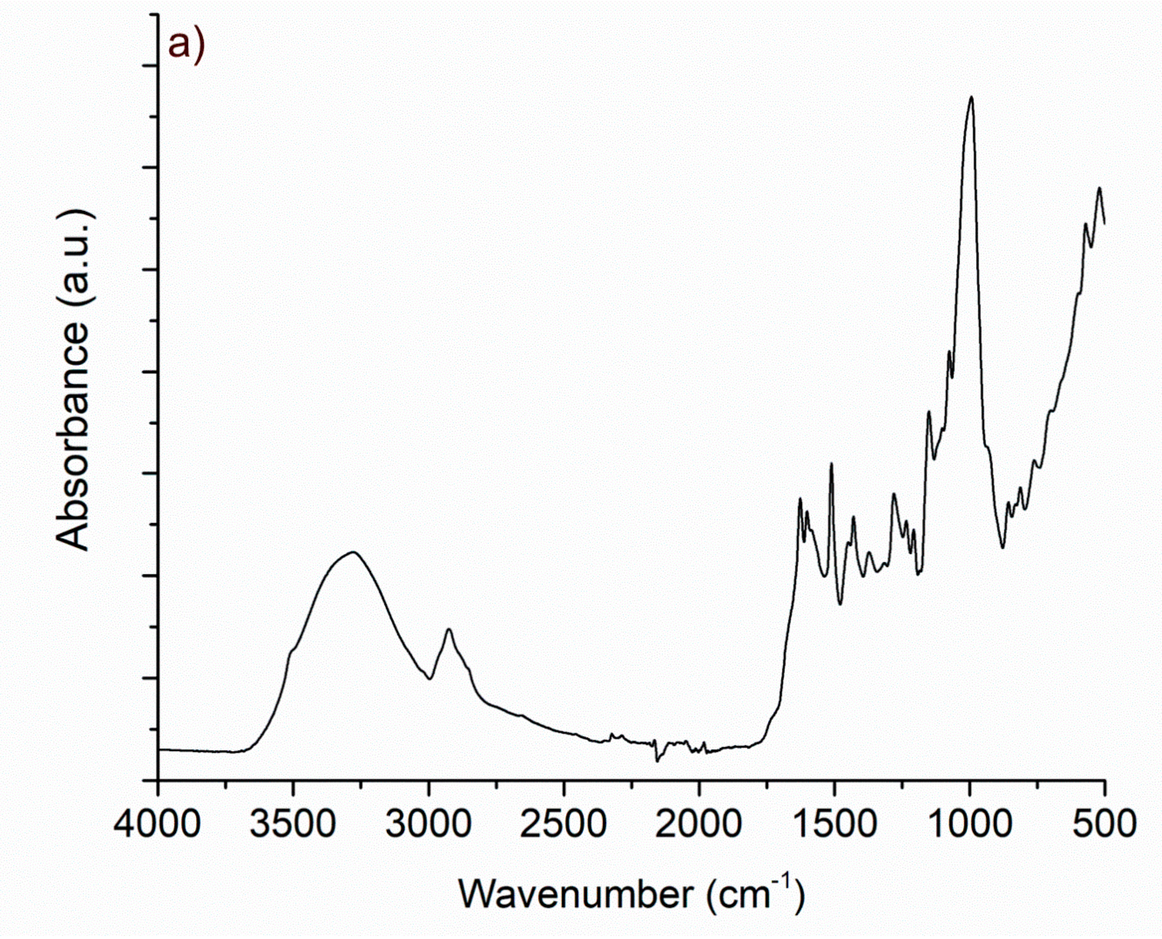

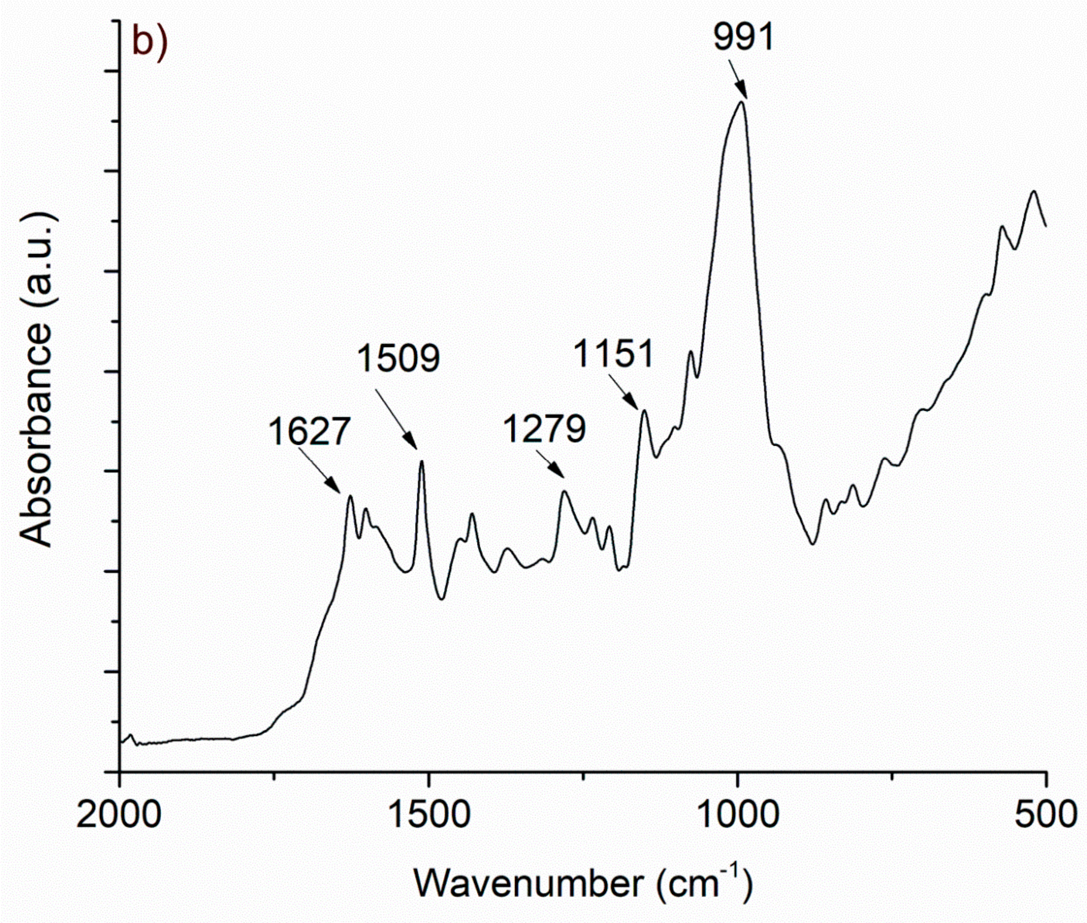

Figure S1. FTIR spectrum of turmeric powder: a) wavenumber 500-4000 cm^{-1} and b) 500-2000 cm^{-1}.

The band at 1509 cm^{-1} could be assigned to curcumin (C=C stretching vibration) The band at 1627 cm^{-1} could be assigned C=O stretching vibration of curcumin molecule. (Elakkiya, 2014; Lv, 2022). These bands are present in the FTIR spectra of composites P1f-P3f.
